# Application of the Theoretical Framework of Acceptability in Health Research: A Scoping Review

**DOI:** 10.1111/jep.70698

**Published:** 2026-10-27

**Authors:** Kayode Olabanji, Marian Kombat, Philip Abutu, Tzeyu Michaud, Chad Abresch

**Affiliations:** ^1^ Department of Health Promotion College of Public Health, University of Nebraska Medical Center Omaha Nebraska USA

**Keywords:** acceptability, health interventions, implementation science, patient and public perspectives, target populations, Theoretical Framework of Acceptability

## Abstract

**Background:**

The Theoretical Framework of Acceptability (TFA) conceptualizes acceptability as a multi‐component construct reflecting anticipated and experienced cognitive and emotional responses to health interventions. Since its introduction in 2017, the TFA has been widely applied, but how it is applied in practice has not been systematically examined.

**Methods:**

We conducted a scoping review of peer‐reviewed studies published between 2017 and 2025 that applied the TFA in the fields of health and healthcare. We searched PubMed, Scopus, and Embase. Data were extracted on study characteristics, construct operationalization, framework application, target population, implementation stage, and proposed extensions. Findings were synthesized descriptively.

**Results:**

We identified 944 records, removed 505 duplicates, and included 303 studies in the final synthesis, with 57% published between 2024 and 2025. All seven TFA constructs appeared in the literature, though unevenly. Affective attitude (98.7%), perceived effectiveness (97.4%), and burden (95.4%) were applied most frequently, while ethicality (83.2%) and opportunity costs (84.5%) were reported less often. Applications were concentrated in retrospective assessments (52.8%) and during implementation (52.1%). Patient‐only populations accounted for 30.4% of studies; however, 35.3% of studies assessed acceptability across two or more target population groups. Thirty‐six studies (11.9%) proposed additional acceptability dimensions, spanning relational, cultural and contextual, and economic considerations.

**Conclusions:**

The TFA has been rapidly adopted in health and healthcare research, but important differences remain in how acceptability is assessed across studies. Proposed relational, cultural or contextual fit, and economic dimensions highlight potential areas for future refinement of the framework.

## 1 Introduction

Despite longstanding recognition of acceptability as a core determinant of intervention success, early implementation research treated the construct inconsistently and with limited conceptual clarity. Acceptability appeared across feasibility studies, pilot evaluations, and process evaluations, often used interchangeably with satisfaction, uptake, adherence, and appropriateness, with little agreement on what distinguished it from related constructs or how it should be operationalized [1, 2]. As a result, the field lacked a shared understanding of what acceptability represented, whose experiences it captured, or how it related to other implementation outcomes [3, 4].

Important structure was introduced by the identification of acceptability as a distinct implementation outcome [2] However, this classification positioned acceptability analytically without theorizing the construct itself. Other commonly used frameworks addressed related questions but did not conceptualize acceptability as an experiential phenomenon. For instance, technology acceptance frameworks emphasized behavioral intention rather than cognitive and emotional responses to interventions, while the Theoretical Domains Framework focused on determinants of behavior rather than subjective appraisal [5, 6, 7]. This limitation became more consequential as major health governance bodies, including the World Health Organization and the Advisory Committee on Immunization Practices, began formally incorporating acceptability as a criterion alongside effectiveness, feasibility, and equity in health system decision making and recommendations [8, 9, 10]. Pragmatic instruments such as the Acceptability of Intervention Measure (AIM) were also developed to operationalize acceptability as an implementation outcome, but they did not articulate the mechanisms or processes through which acceptability is formed or experienced [11].

The Theoretical Framework of Acceptability (TFA) was developed to address this gap, defining acceptability as a multifaceted construct reflecting the extent to which people delivering or receiving a healthcare intervention consider it appropriate based on their anticipated or experienced cognitive and emotional responses [4] The framework treats acceptability as subjective and dynamic and as varying across target populations, contexts, and stages of implementation. It identifies seven component constructs including affective attitude, burden, ethicality, intervention coherence, opportunity costs, perceived effectiveness, and self‐efficacy, which together describe different ways in which acceptability may be assessed.

Since its introduction, the framework has been applied across a wide range of health intervention contexts, and efforts have been made to operationalize its constructs through both quantitative and qualitative approaches, including theory‐informed questionnaires, interview guides, and analytic coding frameworks [12, 13]. However, empirical studies have reported challenges related to construct salience, comprehension overlap, and context‐driven adaptation [12, 13, 14, 15, 16], suggesting that while the framework has provided a shared language for acceptability, its operationalization remains heterogeneous across settings. Such variation is consistent with the framework's intended adaptability, although clearer guidance for construct operationalization and reporting may support greater transparency and comparability across studies.

This scoping review asks how the TFA has been applied and operationalized in health research among individuals delivering or receiving health interventions. Specifically, first, in what study designs has the TFA been applied? Second, how have the seven core TFA constructs been operationalized, and which of these constructs are most frequently not reported or incompletely reported? Third, which target populations have been represented in acceptability assessments? Fourth, what constructs have been proposed beyond the TFA's original seven domains, and what do these suggest about the framework's theoretical boundaries?

## 2 Methods

### 2.1 Study Design

We conducted a scoping review to map how the TFA has been applied in health intervention research and to identify patterns in the utilization and reporting of its constructs. The review followed the methodological framework proposed by Arksey and O'Malley [17] (identifying the research question, identifying relevant studies, study selection, charting the data, and collating, summarizing, and reporting results). Hence, our study is largely descriptive and follows the Preferred Reporting Items for Systematic Reviews and Meta‐Analyses extension for Scoping Reviews (PRISMA‐ScR). A protocol was not registered in advance, but a completed PRISMA‐ScR checklist is provided as a Supplementary file S4 [18, 19].

### 2.2 Search Strategy

We searched PubMed, Scopus, and Embase for English‐language peer‐reviewed articles published between 2017 and 2025. The start date corresponds to the year the TFA was first published. The same conceptual search string was used across all three databases: “theoretical framework of acceptability” AND (health OR healthcare OR medical OR clinical) AND (intervention OR treatment OR service OR program OR policy). Database‐specific syntax was applied where required. Scopus searches were limited to title, abstract, and keyword fields using the TITLE‐ABS‐KEY tag, and Embase searches used equivalent field restrictions alongside publication year and language limits. The literature searches were conducted on December 16, 2025.

### 2.3 Eligibility Criteria

We included studies that used TFA, in whole or in part, to conceptualize, guide data collection on, or analyze the acceptability of a health intervention, program, service, treatment, or policy, provided they reported findings relevant to at least one TFA construct. We placed no restriction on study design or geographic setting.

Studies were excluded at the title‐screening stage if they made no reference to acceptability. Abstract and full‐text eligibility was subsequently assessed using the following exclusion criteria. First, papers that did not use the TFA in any capacity were excluded regardless of whether acceptability was otherwise assessed. Second, studies that used the TFA solely to develop or psychometrically validate a measurement instrument, without reporting construct‐level acceptability findings about health interventions, were excluded. Third, papers that applied the TFA but reported no findings attributable to at least one construct were excluded. Fourth, conference abstracts, posters, and symposium proceedings were excluded on the basis that insufficient methodological detail was available for reliable coding and full‐text review was not possible. Fifth, study protocols and registered trials were excluded. Sixth, papers published in 2026 or later fell outside the temporal scope of the review and were therefore excluded. Finally, where multiple papers drew on the same ethics approval, cohort, or research program without presenting a meaningfully distinct acceptability question, target population group, or longitudinal time point, we retained the most methodologically complete paper and excluded the remaining entries.

### 2.4 Article Selection

Records retrieved from the three databases were imported into Rayyan for deduplication and title/abstract screening. Following duplicate removal, one reviewer screened all remaining records against the eligibility criteria. Records that did not meet the inclusion criteria were excluded, and the remaining articles were retrieved for full‐text assessment. One reviewer then evaluated all full‐text articles for eligibility, documenting reasons for exclusion. A complete list of full‐text exclusions and reasons is provided in Supplementary File S3.

### 2.5 Data Extraction

A structured data extraction form was developed in Microsoft Excel, and one reviewer extracted data from all included studies using a standardized codebook (Table 1; Supplementary File S1). Data were collected across five domains: study characteristics; intervention; TFA application; implementation stage and timing; and target population and methodological characteristics. Because this review sought to characterize how the TFA has been applied in practice, implementation stage and timing of assessment were treated as distinct variables. Implementation stage reflected the operational status of the intervention at the time of evaluation, whereas timing captured the participant's temporal relationship to the intervention experience.

**Table 1:** Data extraction codebook summary.

Variable	Operationalization
A. Study characteristics	
Study design	Overall methodological approach (qualitative, quantitative, or mixed methods).
Data collection	Primary method(s) used to collect acceptability data.
Sample size (*N*)	Total study sample size or acceptability‐specific subsample, where applicable.
B. TFA application characteristics	
TFA application	Primary, supporting, or post‐hoc application.
Framework modified	Explicit modification of TFA structure
Acceptability scope	Sole focus or part of broader evaluation
C. TFA construct operationalization	
Affective attitude	How the individual feels about the intervention.
Burden	Perceived effort required to participate in or receive the intervention.
Ethicality	Extent to which the intervention fits with the individual's value system.
Intervention coherence	Extent to which the participant understands the intervention and how it works.
Opportunity costs	Benefits or values that must be given up engaging with the intervention.
Perceived effectiveness	Perceived likelihood that the intervention will achieve its purpose.
Self‐efficacy	Confidence that participants can perform the behaviors required to engage.
D. Implementation characteristics	
Implementation stage	Development, pre‐, during, or post‐implementation
Timing of assessment	Prospective, concurrent, retrospective, or longitudinal
Implementation setting	Delivery setting or context
E. Target population characteristics	
Target population	The individual or group whose acceptability of the intervention was assessed
Number of target population groups	Number of distinct target population categories included within a study.
F. Framework extensions	
Emerging constructs	Additional acceptability dimensions explicitly proposed by study authors as extending, challenging, or addressing limitations of the original seven TFA constructs. Constructs had to represent target population appraisals rather than solely contextual or structural conditions.
G. Intervention	
Type of intervention	The category of intervention that was the object of acceptability evaluation, coded into nine groups (treatment, prevention, screening, service delivery, technology, training/education, implementation strategy, policy, and other). Full category definitions are provided in the codebook.

To determine which TFA construct was assessed, a four‐pathway decision rule was applied. A construct was coded as present when it was:

1. Explicitly named or described in the results or discussion;
2. Operationalized through a reported instrument, survey, topic guide, or appendix;
3. Clearly reflected in reported findings despite not being labeled using TFA terminology; or
4. Documented in the Methods through an analytic matrix, codebook, or similar approach.

Constructs were coded as unclear when they could reasonably be inferred but insufficient supporting evidence was available, such as when authors stated that the TFA informed instrument development without providing additional detail. Constructs were coded as ‘No’ when authors explicitly stated that the construct was not assessed or when no evidence of its inclusion could be identified.

In addition to the seven established TFA constructs, we recorded constructs that study authors proposed as extending or addressing gaps within the framework. To qualify, a construct had to:

1. Represent an appraisal of the intervention by a target population rather than an external contextual factor;
2. Be explicitly positioned by the authors as a gap in, or addition to, the TFA.

For constructs that were not reported, reasons for non‐reporting were also documented and categorized as:

1. Never elicited, where the construct was not included in data collection instruments;
2. Post hoc gap, where the TFA was applied retrospectively to data not originally collected to assess the construct;
3. Did not emerge, where the construct was available for elicitation but no related findings were reported; or
4. Not applicable, where authors explicitly indicated that the construct was not relevant to the intervention context.

To assess extraction reliability, two reviewers independently examined random samples of the extracted data. Disagreements were resolved through discussion and consensus. Reviewer 1 assessed 30 studies (10%) and flagged some entries for discussion. Resolution of these discrepancies resulted in 25 coding changes, primarily relating to implementation setting and the classification of multicomponent interventions. Reviewer 2 independently assessed 10 studies (3.3%) and had two questions that were resolved after a discussion, as these differences reflected alternative interpretations rather than coding errors. The full extraction sheet is available as Supplementary file S2.

### 2.6 Data Synthesis

We synthesized data descriptively, calculating frequency distributions across categorical variables such as publication year, intervention type, implementation setting, study design, target population category, implementation stage, timing of assessment, and TFA construct usage. We also examined patterns in construct operationalization and emerging acceptability dimensions beyond the seven core TFA constructs [17, 19, 20]. This involved coding of relevant texts extracted from included studies, development of descriptive themes, and grouping of themes into analytic clusters representing patterns across the literature [17, 18, 20]. For studies proposing acceptability dimensions beyond the seven core TFA constructs, all author‐labeled constructs were extracted verbatim. These constructs were then inductively grouped by the reviewer into thematic clusters based on conceptual similarity.

### 2.7 Quality Appraisal

Formal quality appraisal of included studies was not conducted as the primary aim of this review was to map the extent and nature of TFA application in health intervention research. Study design and data collection characteristics are nonetheless reported descriptively as part of the synthesis, which allows readers to consider methodological differences when interpreting our findings.

## 3 Results

Database searches identified 944 records across PubMed, Scopus, and Embase. After removal of 505 duplicate records, 439 titles and abstracts were screened. Full‐text articles were sought for 389 records, of which 384 were retrieved and assessed for eligibility. A total of 303 studies met the inclusion criteria and were included in the review (Figure 1).

**Figure 1:**
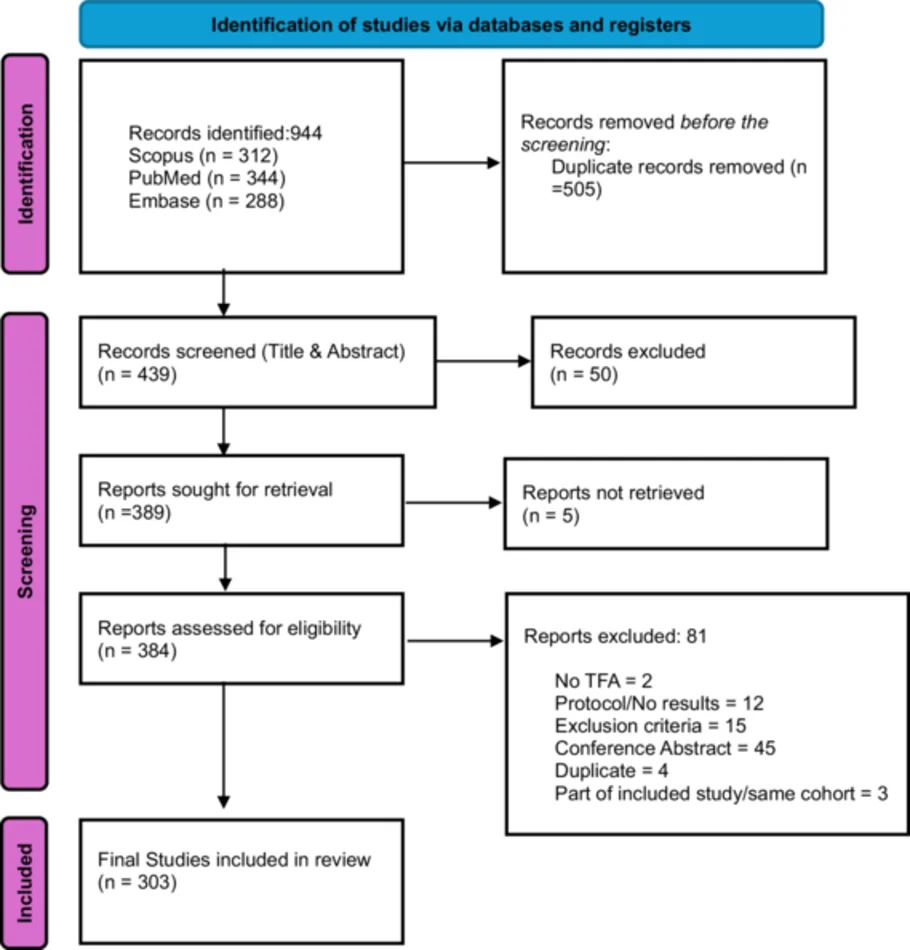
Study selection flow diagram.

### 3.1 Study Characteristics

The included primary studies collectively engaged more than 28,000 participants across diverse geographic settings. Publication volume increased markedly over the review period, with more than half of all included studies (57.1%) published in 2024 or 2025. Detailed study characteristics are presented in Table 2.

**Table 2:** Included study characteristics.

Characteristics	*N* (%)
Publication year	
2019	3 (1%)
2020	10 (3.3%)
2021	23 (7.6%)
2022	47 (15.5%)
2023	47 (15.5%)
2024	65 (21.5%)
2025	108 (35.6%)
Type of intervention	
Treatment	80 (26.4%)
Service delivery	56 (18.5%)
Technology	45 (14.9%)
Prevention	37 (12.2%)
Screening	33 (10.9%)
Training/Education	30 (9.9%)
Implementation Strategy	14 (4.6%)
Policy	4 (1.3%)
Other	4 (1.3%)
Sample size	
1–20	99 (32.7%)
21–50	117 (38.6%)
51–100	43 (14.2%)
101–250	25 (8.3%)
251–500	7 (2.3%)
501–1000	7 (2.3%)
> 1000	5 (1.7%)

*Note:* Percentages may not total 100 due to rounding.

### 3.2 How TFA Constructs Were Operationalized

The TFA was used as the primary framework in most studies (67.0%), with a further quarter applying it post‐hoc; modifications to the framework were rare (5%). Acceptability assessments were predominantly retrospective and concentrated during active implementation. Most studies employed qualitative designs (58.7%), followed by mixed‐methods (27.7%) and quantitative approaches (13.5%). Interviews were the most common data collection method (45.5%). Acceptability was assessed as a standalone outcome in more than two‐thirds of studies (68.6%), whereas 31.4% examined acceptability alongside other outcomes or frameworks, most commonly feasibility. Each of the seven TFA constructs was operationalized across the included studies, although their frequency of use varied considerably. Affective attitude (98.7%), perceived effectiveness (97.4%), and burden (95.4%) were applied most frequently, whereas ethicality (83.2%) and opportunity costs (84.5%) were the least reported.

Most studies (64.7%) examined acceptability in a single target population group, while 25.4% included two groups and 9.9% included three or more groups. Patients were the most frequently studied single target population category (92 studies, 30.4% of all studies), followed by providers (12.2%) and service users (9.6%). Overall, 107 studies (35.3%) assessed acceptability across more than one target population group, exceeding the frequency of any individual target population category. Administrators were rarely represented, appearing in only one study (0.3%). Full methodological characteristics are summarized in Table 3.

**Table 3:** Overview of TFA utilization in the included studies (*n* = 303).

	*N* (%)
Study Design	
Qualitative	178 (58.7%)
Mixed methods	84 (27.7%)
Quantitative	41 (13.5%)
Data collection method	
Interviews only	138 (45.5%)
Surveys only	52 (17.2%)
Surveys and interviews	47 (15.5%)
Focus groups and interviews	39 (12.9%)
Focus groups only	13 (4.3%)
Focus groups and surveys	4 (1.3%)
Focus groups, interviews, and surveys	2 (0.7%)
Observation and interviews	3 (1%)
Other	5 (1.7%)
Assessment scope	
Acceptability only	208 (68.6%)
Acceptability + other outcomes/frameworks	95 (31.4%)
Implementation stage	
During implementation	158 (52.1%)
Pre‐implementation	62 (20.5%)
Post‐implementation	59 (19.5%)
Development/design	24 (7.9%)
Timing of assessment	
Retrospective	160 (52.8%)
Prospective	69 (22.8%)
Concurrent	51 (16.8%)
Longitudinal	23 (7.6%)
Implementation setting	
Hospital	84 (27.7%)
Community	77 (25.4%)
Digital/remote	45 (14.9%)
Primary care	40 (13.2%)
Home	25 (8.3%)
Other	16 (5.3%)
School	11 (3.6%)
Custodial facility	5 (1.7%)
Target population category	
Multiple groups	101 (33.3%)
Patients	92 (30.4%)
Providers	37 (12.2%)
Service users	29 (9.6%)
Community	17 (5.6%)
Healthcare workers	17 (5.6%)
Caregivers	7 (2.3%)
Volunteers/lay deliverers	2 (0.7%)
Administrators	1 (0.3%)
Number of target population groups per study	
One group	196 (64.7%)
Two groups	77 (25.4%)
Three or more groups	30 (9.9%)
How TFA was applied	
Primary framework	203 (67.0%)
Post‐hoc	79 (26.1%)
Supporting framework	21 (6.9%)
Framework modification	
Not modified	288 (95%)
Modified	15 (5%)

Coverage of seven core TFA constructs			
Construct	Yes (%)	No (%)	Unclear (%)
Affective attitude	299 (98.7%)	2 (0.7%)	2 (0.7%)
Perceived effectiveness	295 (97.4%)	6 (2.0%)	2 (0.7%)
Burden	289 (95.4%)	12 (4.0%)	2 (0.7%)
Self‐efficacy	282 (93.1%)	18 (5.9%)	3 (1.0%)
Intervention coherence	279 (92.1%)	22 (7.3%)	2 (0.7%)
Opportunity costs	256 (84.5%)	37 (12.2%)	10 (3.3%)
Ethicality	252 (83.2%)	41 (13.5%)	10 (3.3%)

*Note:* 1. Percentages may not total 100 due to rounding. 2. Target population category records the type of population engaged, while Number of target population groups per study numbers distinct subgroups sampled. A study in a single category may still comprise two or more subgroups (e.g., Providers containing surgeons and surgical trainees), so the counts differ by design.

Ethicality and opportunity costs were the constructs most frequently “not reported.” An overview of reasons for non‐reporting by construct is provided in Table 4.

**Table 4:** Frequency and reasons for not reporting the seven core TFA constructs in the included studies where determinable.

Construct	Not reported, *n*	Primary reason (#of studies)
Opportunity costs	22	Never elicited [21, 22, 23, 24, 25, 26, 27, 28, 29] Did not emerge [30, 31, 32, 33, 34, 35, 36, 37, 38, 39] Not Applicable [40, 41] Post‐hoc gap [42]
Ethicality	17	Never elicited [21, 22, 23, 26, 43, 44, 45, 46, 47, 48, 49] Did not emerge [30, 32, 39] Post‐hoc gap [50, 51, 52]
Intervention coherence	12	Never elicited [21, 23, 24, 25, 45] Did not emerge [34] Post‐hoc gap [42, 53] Not applicable [40, 54, 55, 56]
Burden	7	Did not emerge [31] Post‐hoc gap [42, 53] Never elicited [21, 26, 27] Not applicable [57]
Self‐efficacy	6	Never elicited [21, 26, 28, 58] Post‐hoc gap [53, 59]
Perceived effectiveness	4	Never elicited [21, 25, 28] Post‐hoc gap [53]
Affective attitude	1	Post‐hoc gap [53]

*Note:* Counts include only those studies for which a reason for non‐reporting could be determined from the published report. Totals therefore differ from the No counts in Table 3.

### 3.3 Emerging Acceptability Constructs Beyond the TFA Seven

Thirty‐six studies (11.9%) proposed one or more acceptability constructs extending beyond the seven core TFA domains. Through these studies, researchers introduced constructs descriptively grouped into ten thematic clusters. The most frequently occurring themes related to cultural and contextual fit (*n* = 7) and trust and relational factors (*n* = 5). Other proposed dimensions focused on perceived benefits, safety and risk, accessibility and usability, personal relevance, social and structural factors, system‐level considerations, economic and practical issues, and patient role, identity, and privacy. Full cluster details are presented in Table 5 below.

**Table 5:** Emerging constructs identified in the included studies.

Theme	*n*	Emerging construct
Trust and relational factors	5	Trust [60] Alliance (therapeutic working relationship) [32] Client‐provider relationship [61] Trust in intervention deliverer [33] Trust in doctors [62]
Cultural and contextual fit	7	Cultural sensitivity [63] Cultural inclusivity [64] Contextual factors [65, 66, 67] Organizational context [68] Design and Delivery contexts [69]
Safety and risk perception	4	Perceived safety and risk [70] Safety and risk [71] Perceived Safety [72] Perceived Negative Effects [15]
Accessibility and usability	4	Accessibility [73] Perceived Ease of Use [53] Usability [67] Facility fit [74]
Perceived need and relevance	4	Perceived need [75, 76] Content Relevance [77] Relevance [15] Timing of intervention [75] Personal context [76]
Social and structural factors	3	Stigma [78] Structural stigma [79] Equity [78] Perceived Social Acceptability [15]
Perceived benefit and effectiveness extensions	5	Perceived Benefits [80] Information gain [81] Perceived Positive Effects [15] Accountability [82] General Acceptability [83]
Integration and system‐level factors	3	Integration with health services [84] Flexibility [67] Multimorbidity [85]
Economic and practical considerations (not opportunity cost)	3	Willingness to pay [58] Knowledge gap [86] Product Attributes [87]
Patient role, identity and privacy	3	Participant role [88] Appropriateness [88] Perceived appropriateness [89] Privacy [90]

*Note:* The headline total counts distinct studies (36; 11.9% of *n* = 303). Thematic clusters sum to 41 because studies proposing constructs across more than one theme are counted in each; Casale et al. (2023) appears in four clusters and Hardeman et al. (2025) in three. Per‐theme percentages (over *n* = 303) therefore sum to more than 11.9%.

## 4 Discussion

This scoping review examined how the Theoretical Framework of Acceptability has been applied in health research since its introduction in 2017, with particular attention to construct operationalization, target populations, and timing of acceptability assessment.

Drawing on 303 studies, the findings show that while the TFA has been widely taken up to structure acceptability evaluations, its empirical application remains uneven. Studies tended to concentrate on a subset of constructs, focus predominantly on patient perspectives, and assess acceptability retrospectively during implementation. At the same time, recurring proposals for additional dimensions of acceptability suggest challenges in operationalizing certain constructs and emerging tensions at the boundaries of the framework. Together, these patterns highlight how the TFA is functioning in practice and suggest several priorities for future research. Variation in construct coverage may partly reflect the dynamic nature of acceptability, as some constructs appear more relevant at different stages of intervention development and implementation. This is especially evident in post‐hoc applications [88, 91, 92], where available data may not support assessment of all seven constructs. Clearer guidance on construct operationalization, broader inclusion of organizational and system‐level target populations, and further examination of recurring relational, cultural, contextual, and economic dimensions may strengthen future applications of the framework.

### 4.1 Use of the TFA

One of the central aims of this review was to characterize how the TFA has been applied in empirical studies, including patterns of framework use, construct operationalization, target population inclusion, and timing of assessment. The predominance of studies using the TFA as a primary framework for assessing acceptability suggests that researchers increasingly view acceptability as a distinct outcome worthy of direct assessment. Beyond its primary role, the TFA also appeared as a supporting framework alongside other theoretical lenses and was applied post hoc as an analytic tool in a substantial proportion of studies, both of which speak to its versatility. However, post hoc application can limit which constructs are captured and how comprehensively acceptability is represented.

A notable finding was that nearly 70% of studies assessed acceptability as a standalone outcome rather than as one component of a broader evaluation. This pattern is consistent with the TFA's original aim of providing a dedicated conceptual framework for understanding acceptability. Nevertheless, the continued co‐assessment of acceptability with feasibility, usability, adherence, and behavior change outcomes indicates that acceptability remains closely connected to broader implementation and intervention evaluation efforts.

### 4.2 Operationalization of the Seven Core Constructs

Affective attitude, perceived effectiveness, and burden were assessed in nearly all studies. As a group, these three constructs reflect dimensions of acceptability that are relatively easy for participants to articulate through questions about their experiences, perceived benefits, and required effort [4, 93]. By contrast, ethicality and opportunity costs were the constructs most frequently not reported. Both require participants to engage in more complex forms of reasoning. Ethicality involves normative judgments about whether an intervention aligns with personal or cultural values, while opportunity costs require reasoning about counterfactual trade‐offs, that is, what must be given up in order to engage with the intervention [13, 94]. These are dimensions unlikely to surface without deliberate prompting.

Where the reasons for non‐reporting could be determined, they pointed to methodological constraints rather than theoretical disagreement. Four categories of non‐reporting were identified across the literature. In the largest category, never elicited, constructs were not included in the data collection instrument or topic guide, reflecting limitations in instrument design [95, 96]; did not emerge, constructs were elicited but did not arise in participant responses [30, 31]; post‐hoc analytic gap, the TFA was cited as the analytical framework, but specific constructs were not coded during post‐hoc analysis [14, 32], rather than participant data being absent; not applicable, the construct was determined not to be relevant to the intervention or population under study [61, 97]. Non‐reporting is not inherently problematic, but authors should clearly report why constructs were not included. This distinction helps differentiate deliberate study scope decisions from methodological gaps.

Taken together, acceptability risks being assessed only partially in practice, even when it is theoretically well defined. Although the TFA offers comprehensive conceptual coverage, its empirical application depends on whether studies are designed to elicit the full range of acceptability constructs rather than only those most readily accessible to the target population. The uneven operationalization of some constructs may also indicate that different dimensions of acceptability vary in salience across intervention contexts and implementation stages, highlighting the dynamic nature of acceptability as an outcome. Future advances in both the framework and its application are therefore likely to depend on clearer guidance for construct‐level elicitation, measurement, and reporting. Where revisions or extensions to the framework are proposed, they should be developed with the same theoretical rigor and methodological transparency as the original framework and viewed as refinements rather than departures from the TFA. It may also be helpful for the TFA authors [4] to review and build a refinement in line with the original design.

### 4.3 Perspectives of Target Populations

Another key objective of the review was to examine the target populations whose acceptability was assessed. The TFA defines acceptability as experienced by both those delivering and those receiving interventions, yet the findings reveal a pronounced asymmetry in how this premise is operationalized in practice. On the recipient side, patients and service users together accounted for the majority of single‐population studies, reflecting a strong emphasis on experiential appraisal from the perspective of those the intervention is designed for. In contrast, providers, healthcare workers, caregivers, administrators, and lay intervention deliverers were comparatively underrepresented as target populations, despite the influence these groups may have on intervention implementation and sustainability. [97] Administrator perspectives were represented in only one study, limiting conclusions regarding how intervention acceptability is evaluated at leadership and organizational levels. This distinction matters because leadership support and organizational fit are widely recognized as critical to implementation success, and the absence of data should not be read as absence of support.

A related but distinct pattern concerns how the TFA is applied. Where the framework is built into study design from the outset, it shapes what questions are asked, which constructs are elicited, and which target populations are included. When the TFA is applied post hoc to data collected for other purposes, construct coverage is often incomplete, and the range of target populations is limited by the original study design. The predominance of single‐population, patient‐focused designs may reflect both deliberate scope decisions and, in post‐hoc applications, structural limitations of what the available data can support. Expanding assessments to include providers, administrators, caregivers, and system‐level actors would not dilute the framework's focus but rather bring empirical practice into closer alignment with its original premise that acceptability is experienced by those delivering as well as those receiving interventions.

### 4.4 Timing and Implementation Stage of Assessment

Most studies assessed acceptability during active implementation, with fewer conducted pre‐implementation, post‐implementation, or during intervention development. These patterns are not independent of study design. Implementation stage and the timing of data collection reflect how a study is constructed rather than free‐standing methodological choices. Retrospective assessment is common in qualitative and post‐hoc TFA applications where data were gathered during or after implementation, while prospective and longitudinal approaches tend to appear in studies designed from the outset to track acceptability across time, such as feasibility trials or pilot studies that embed acceptability as a primary outcome. The predominance of qualitative study designs and post hoc applications likely contributed to the frequent use of retrospective assessments. What the data do show is that acceptability has been assessed more often once participants have lived experience of an intervention than before or across its implementation. Whether this represents a limitation depends on the research question. Retrospective assessment captures experienced acceptability with depth and contextual richness, while prospective assessment, such as in feasibility or pilot phases before wider rollout, can inform design decisions before intervention features are fixed [4, 98]. Longitudinal designs, which remain rare, offer a distinct contribution by capturing how acceptability shifts over time, a dimension the TFA explicitly theorizes as important but which the empirical literature has not yet examined systematically.

### 4.5 Proposed Extensions and Theoretical Boundaries

A notable finding of this review was that 36 studies proposed one or more constructs extending beyond the TFA seven. While many of these overlapped with existing constructs and are best understood as reflecting variations in operationalization or context‐specific adaptations, several thematic clusters point to what might be called theoretical boundary questions. These recurring proposals raise the possibility that some acceptability judgments may not be fully captured by the existing seven and may warrant consideration as additional construct domains. Trust and relational factors were proposed by five studies, with constructs including trust, therapeutic alliance, and client‐provider relationship [32, 33, 60, 61, 62]. Seven studies proposed constructs related to cultural and contextual fit, including cultural sensitivity and organizational context [63, 64, 65, 66, 67, 68, 69]. Three studies raised constructs operating at a social and structural level, including equity and structural stigma [15, 78, 79]. Three studies introduced constructs with economic or practical dimensions, including willingness to pay and product attributes [58, 86, 87]. Their recurring presence across diverse health sectors and populations suggests that in certain implementation contexts, particularly those involving long‐term relationships, socially sensitive settings, or system‐level decision making, the existing seven constructs may not fully account for what shapes acceptability judgments.

The case for treating some of these dimensions as distinct is most evident when acceptability is considered beyond individual recipients. Decisions about adoption and sustainability are often made by actors such as payers, policymakers, and system leaders, for whom acceptability is shaped by economic value and cost‐effectiveness, and organizational feasibility. The existing constructs of opportunity costs and perceived effectiveness offer partial coverage of these considerations, but the included studies suggest they are not always sufficient to capture how acceptability is formed at an organizational or system level [58, 86, 87]. This does not necessarily mean new constructs are required. Many of the proposed extensions may be best handled through more deliberate operationalization of existing domains, or through clear reporting of how context‐specific dimensions were treated within the existing framework. However, stronger consideration is warranted when a proposed dimension appears consistently across settings, is not adequately captured by existing constructs, and reflects a distinct form of acceptability appraisal (e.g., trust, cost effectiveness, and context). Any such development would need to operate at a clearly specified target population level and remain anchored in acceptability as an evaluative judgment, so that new constructs extend the framework's coverage rather than diffusing its conceptual focus.

### 4.6 Strengths and Limitations

This review has several strengths. It represents a comprehensive mapping of the empirical application of the TFA since its introduction, drawing on a large and recently published body of research. The use of explicit, construct‐level decision rules allowed for consistent and transparent identification of how each TFA domain was operationalized, accommodating variation in study design and reporting practices while maintaining theoretical fidelity. By examining acceptability across implementation stages, timing of assessment, and target population groups, the review extends beyond simple frequency mapping to illuminate how acceptability is positioned and mobilized in practice. Together, these features enhance the interpretability and usefulness of the findings for both empirical researchers and those engaged in theoretical development within implementation science.

Several limitations should be considered when interpreting the findings. First, the identification of TFA constructs relied on how authors described their data and methods; where reporting was incomplete or ambiguous, construct classification depended on interpretive judgment applied through structured decision rules, meaning the findings reflect the state of reporting in the literature rather than necessarily the full scope of constructs operationalized in practice. Second, screening and full‐text assessment were conducted by a single reviewer, which deviates from the two‐reviewer minimum recommended in the JBI methodological guidance [99] To mitigate this, a subset of extractions was independently audited: MK audited 30 studies (10%) and PA audited 10 studies (3.3%), with discrepancies resolved by discussion. Finally, as with all reviews of published literature, the findings are subject to publication bias, with studies reporting low acceptability, implementation challenges, or framework limitations likely underrepresented.

### 4.7 Future Directions

Future research should prioritize more systematic and transparent operationalization or exclusion of all seven TFA constructs, particularly ethicality and opportunity costs, which appear underrepresented due to methodological rather than conceptual constraints. Secondly, greater use of prospective and longitudinal designs would better align empirical practice with the framework's dynamic conceptualization of acceptability. Expanding assessments beyond patients and providers to include administrators, payers, and other system‐level actors would strengthen the framework's relevance across the health value chain. Finally, theoretical work should focus on clarifying when relational, cultural, and economic dimensions are best integrated within existing constructs and when disciplined construct development is warranted, with explicit attention to target population level.

## 5 Conclusion

This scoping review mapped how the Theoretical Framework of Acceptability has been applied in health research since its introduction, documenting patterns in construct operationalization, target population focus, timing of assessment, and proposed extensions. The findings show that the TFA has achieved broad uptake and improved conceptual clarity around acceptability, while also revealing heterogeneity in empirical application. Persistent challenges include partial use of core constructs, limited representation of non‐patient target populations, and underutilization of prospective and longitudinal designs. Overall, these findings suggest that strengthening acceptability research will depend on clearer operationalization, evidence‐informed theoretical development, and explicit attention to how acceptability operates across different target populations within the health system.

## Author Contributions

Conceptualization: Kayode Olabanji, Chad Abresch; Data Curation: Kayode Olabanji; Methodology: Kayode Olabanji, Tzeyu Michaud, Chad Abresch; Investigation (screening, full‐text assessment, data extraction): Kayode Olabanji; Formal analysis: Kayode Olabanji; Validation: Marian Kombat, Philip Abutu, Tzeyu Michaud, Chad Abresch; Writing – original draft: Kayode Olabanji; Writing – review and editing: Marian Kombat, Philip Abutu, Tzeyu Michaud, Chad Abresch; Supervision: Chad Abresch. All authors approved the final submitted version.

## Funding

The authors have nothing to report.

## Conflicts of Interest

The authors declare no conflicts of interest.

## Use of AI

The authors used a generative AI tool to assist with language editing and to critique and refine the framing of selected points during manuscript preparation. All intellectual content, including the review design, screening, data extraction, analysis, and interpretation, was verified by the authors, who take full responsibility for the integrity of the work.

## Supporting information


Supporting File 1

Supporting File 2

Supporting File 3

Supporting File 4

## Data Availability

All data relevant to this review are included within the article and its supplementary materials, including the data extraction resources and reporting checklist. As this review synthesized evidence from previously published studies, no new primary data were collected.
