# Supplementary material for: Application of the Theoretical Framework of Acceptability in Health Research: A Scoping Review
Source: J Eval Clin Pract. 2026 Oct 27;32(7):e70698. doi: 10.1111/jep.70698 (PMC13712922; doi:10.1111/jep.70698)
Supplement: Supplementary file 2 [file JEP-32-0-s001.docx]

**Supplementary file 3 – Exclusion log**

|  | **Author** | **Year** | **Title** | **Exclusion Category** |
| --- | --- | --- | --- | --- |
| 1 | Perez, J. C. | 2025 | A look into telepsychology in the Philippines: an exploratory-cross-sectional research | No TFA |
| 2 | Frizelle, P. et al. | 2024 | The Feasibility of an Online Language Program Delivered Through Music and the Impact of Dosage on Vocabulary Outcomes in Young Children With Down Syndrome | Full Text never retrieved |
| 3 | Ryan, G. | 2023 | Developing and evaluating a parent-child intervention for children with Autism Spectrum Disorder: A feasibility study | Full Text never retrieved |
| 4 | Yunjing, Y. et al. | 2024 | Factors of communication disorders in ICU machinery ventilates patients based on the theoretical framework of acceptability: a phenomenological study | Full Text never retrieved |
| 5 | Hezel, N. et al. | 2024 | Acceptability of Two Perturbation-Based Balance Training Paradigms: Perturbation Treadmill versus Dynamic Stability Training in the Presence of Perturbations | Full Text never retrieved |
| 6 | Arbour, G. et al. | 2024 | Novel Guided Self-Help for the treatment of Binge Eating Disorder: Feasibility, Acceptability, and Preliminary Efficacy | Full Text never retrieved |
| 7 | Twomey R. | 2021 | BREATHE: A Mixed-methods Evaluation of a Virtual Self-management Program for People Living With Long COVID-19 in Alberta | Protocol |
| 8 | Morrison, J et al. | 2025 | Investigating Attitudes to Gynaecological Rapid Access, Cancer Exclusion and Follow Up Location (GRACEFUL Study) | Protocol |
| 9 | Zbukvic, I. et al. | 2025 | Exploring the Implementation of Cognitive Screening in First-Episode Psychosis Settings: The CogScreen Implementation Study | Protocol |
| 10 | Swingwood, E. et al. | 2023 | Mechanical insufflation-exsufflation to promote extubation success in critically ill adults on intensive care: protocol for a randomised controlled feasibility trial | Protocol |
| 11 | Jaguga et al. | 2022 | A Pilot Study to Evaluate the Feasibility & Acceptability of a Peer-mentor Delivered Substance Use Screening and Brief Intervention for Adolescents in Kenya | Protocol |
| 12 | Bates A et al. | 2022 | A Multi-centre, Randomised, Pilot Feasibility Study to Compare the Effectiveness of Eye-movement Desensitisation and Reprocessing Versus Usual Care in the Psychological Recovery of Intensive Care Survivors (EMERALD) | Protocol |
| 13 | Goh Y, S. | 2022 | A Community-based Multi-domain Psychoeducation Self-management Programme (MPSP) for Adults With Major Depressive Disorder in Singapore: A Pilot Randomized Controlled Trial | Protocol |
| 14 | Duncan S et al. | 2023 | RADICAL: A Mixed Methods Study to Assess the Clinical Effectiveness and Acceptability of an Artificial Intelligence Software to Prioritise Chest X-ray Interpretation | Protocol |
| 15 | Shepherd, L. et al | 2024 | A Mixed-methods Acceptability Study of ProACTive, an Early Acceptance and Commitment Therapy Intervention to Help Burns Patients Adjust to Changes to Appearance | Protocol |
| 16 | Audsley et al. | 2024 | Keep Exercising & Stay Steady (KESS): Development and Feasibility of a Digital Health Intervention to Encourage Exercise Maintenance After Fall Prevention Exercise Programmes End | Protocol |
| 17 | Wong, K. et al. | 2023 | CO-STAR: a study protocol for a randomized feasibility study | Protocol |
| 18 | Umaefulam, V. et al. | 2023 | Assessing the feasibility, acceptability, and fidelity of a tele-retinopathy-based intervention to encourage greater attendance to diabetic retinopathy screening in immigrants living with diabetes | Protocol |
| 19 | Marston, C. et al. | 2025 | Caring for carers of people with advanced cancer at hospital discharge (CARENET): A single-arm open label feasibility trial | Does not meet inclusion criteria |
| 20 | Samuelsson, M. et al. | 2025 | The Swedish theoretical framework of acceptability questionnaire: translation, cultural adaptation, and descriptive pilot evaluation | Does not meet inclusion criteria |
| 21 | Jovic, E. et al. | 2024 | Carer-supported home-based exercises designed to target physical activity levels and functional mobility after stroke: a scoping review | Does not meet inclusion criteria |
| 22 | Haydon, H. M. et al. | 2023 | Development and validation of the Digital Health Acceptability Questionnaire | Does not meet inclusion criteria |
| 23 | Bakare, A. A. et al. | 2024 | Let him die in peace': Understanding caregiver's refusal of medical oxygen treatment for children in Nigeria | Does not meet inclusion criteria |
| 24 | Kellogg, T. & Brockett-Walker, C. | 2025 | Animal-Assisted Interventions for Psychological Distress During Prolonged Intensive Care Unit Stay: A Case Report | Does not meet inclusion criteria |
| 25 | Chen, E. et al. | 2022 | Developing and validating a new scale to measure the acceptability of health apps among adolescents | Does not meet inclusion criteria |
| 26 | El-Den, S. et al. | 2024 | Development and validation of measures of pharmacists' acceptability and willingness to screen for perinatal depression | Does not meet inclusion criteria |
| 27 | Laitinen, A. L. et al. | 2022 | The Tasty School model is feasible for food education in primary schools | Does not meet inclusion criteria |
| 28 | Al-Gadi, I. S. et al. | 2024 | The Psychometric Properties of the Type 1 Diabetes Mellitus Screening Acceptability Assessment (DMSA) Scale among General Population | Does not meet inclusion criteria |
| 29 | Goldsmith, L. P. et al. | 2024 | Development of an intervention for reducing infant bathing frequency | Does not meet inclusion criteria |
| 30 | Longoria, D. A. et al. | 2025 | Mental health services for older Hispanics/Latinos: Incorporating culture and context into clinical approaches to care | Does not meet inclusion criteria |
| 31 | Zewude, B. et al. | 2025 | Addressing scabies among street children in Ethiopia: an ethnographic study of acceptable interventions by prospective recipients and deliverers | Does not meet inclusion criteria |
| 32 | Diallo, A.I. et al. | 2022 | Factors associated with the acceptability of government measures to address COVID-19 in Senegal | Does not meet inclusion criteria |
| 33 | Lynnerup, C. et al. | 2022 | Abstracts 8th PCNE working symposium | Conference Abstract |
| 34 | Scharer, J. L. et al. | 2022 | Speaker abstracts | Conference Abstract |
| 35 | Pavlidou, A. et al. | 2022 | Pharmacy practice research meeting conference abstracts 2022 | Conference Abstract |
| 36 | Kaunda-Khangamwa, B. N. et al. | 2025 | Acceptability and feasibility of administering rts,s/as01 malaria vaccine to school-aged children in southern Malawi | Conference Abstract |
| 37 | Huang, S. et al. | 2025 | Acceptability of Sleep Interventions Among Firefighters | Conference Abstract |
| 38 | Crocker, M. et al. | 2025 | Engaging Stakeholders to Design a Wildfire Smoke Action Plan for Children With Asthma | Conference Abstract |
| 39 | Grimshaw, J. | 2025 | Abstracts from the 8th ICPIC 2025 | Conference Abstract |
| 40 | Dinah, C. et al. | 2021 | The acceptability of geographic atrophy injections (AGAIN) study | Conference Abstract |
| 41 | Parhar, K. et al. | 2022 | Treatment of mechanically ventilated patients with hypoxemic respiratory failure | Conference Abstract |
| 42 | Beaulieu, M. C. et al. | 2020 | Mindfulness-based stress reduction (PARE0016) | Conference Abstract |
| 43 | Sharrad, K. et al. | 2022 | Abstracts | Conference Abstract |
| 44 | Montague, S. et al. | 2020 | High patient acceptability for a hepatitis C mobile outreach service | Conference Abstract |
| 45 | Channa K K et al. | 2022 | Exploring elderly patients perspectives on deprescribing: interim analysis | Conference Abstract |
| 46 | Bekker-Jensen, D. et al. | 2025 | Early follow-up and support for survivors following out-of-hospital cardiac arrest | Conference Abstract |
| 47 | Anonymous | 2024 | Exploring the acceptability of Fast Track to Health for adolescents | Conference Abstract |
| 48 | Martin, S. et al. | 2022 | Project BETTER Acceptability | Conference Abstract |
| 49 | Knox, L. et al. | 2022 | Feasibility and acceptability of telehealth in motor neuron disease | Conference Abstract |
| 50 | Pavlidou, A. et al. | 2023 | IDF2022-0718 Feasibility study of an m-health intervention for diabetes, in Cyprus | Conference Abstract |
| 51 | Pavlidou, A. et al. | 2023 | Development and feasibility study of a community pharmacy intervention for type 2 diabetes, in Cyprus | Conference Abstract |
| 52 | Carrion, M. N. et al. | 2023 | How to encourage primary care providers to screen for chagas disease | Conference Abstract |
| 53 | Neumark, S. et al. | 2023 | Translation of novel pharmacogenomic testing | Conference Abstract |
| 54 | Bullock, L. et al. | 2023 | Testing a new prototype osteoporosis shared decision-making intervention (iFraP) | Conference Abstract |
| 55 | Roche, S. et al. | 2023 | Pharmacy-based PREP delivery in Kenya | Conference Abstract |
| 56 | Davis, M. et al. | 2023 | 30th European Congress on Obesity (ECO 2023) | Conference Abstract |
| 57 | Cornwall, N. et al. | 2023 | Acceptability of a prototype practice pharmacist-led proactive review for persistent pain (PROMPPT study) | Conference Abstract |
| 58 | Brown, S. et al. | 2023 | Exploring the acceptability of a community pharmacy-based hypertension visualisation intervention | Conference Abstract |
| 59 | Churchill, V. et al. | 2023 | Evaluation of a theory-informed video intervention using the TFA | Conference Abstract |
| 60 | Jinks, C. et al. | 2024 | Bracing acceptability and adherence for knee osteoarthritis | Conference Abstract |
| 61 | Kapp, S. et al. | 2024 | The 13th World Research Congress of the EAPC | Conference Abstract |
| 62 | Williamson, T. et al. | 2024 | CACPR 2024 Spring Conference Abstracts | Conference Abstract |
| 63 | Thompson, C. et al. | 2024 | Mixed-methods evaluation of acceptability of the tobacco dependency treatment offer for NHS staff | Conference Abstract |
| 64 | Porter, L. et al. | 2024 | Veg Stars for the 3 Rs | Conference Abstract |
| 65 | Tadrous, R. et al. | 2024 | A mixed-method feasibility study of an intervention to reduce sedentary behavior | Conference Abstract |
| 66 | Dixon, G. et al. | 2024 | The role of the endothelial glycocalyx in fibrotic interstitial lung disease | Conference Abstract |
| 67 | Osei-Safo, E. K. et al. | 2025 | Designing a lifestyle intervention to optimize cardiovascular health in high-risk prenatal and postnatal women | Conference Abstract |
| 68 | McIntosh, R. et al. | 2025 | Supporting high-quality nutrition in cancer care with a malnutrition governance toolkit | Conference Abstract |
| 69 | Tan, S. F. & Briley, P. | 2025 | Co-Producing a Survey on Prospective Acceptability of Neuromodulation for Mental Health Conditions with Lived Experience Experts | Conference Abstract |
| 70 | Phillips, K.-A. et al. | 2025 | A process evaluation trial of a telehealth service intervention to support uptake of breast cancer prevention medications | Conference Abstract |
| 71 | Richardson, P. et al. | 2025 | Patient Perceptions of Sustainability in Elective Surgery: A Qualitative Study | Conference Abstract |
| 72 | Watts, J. et al. | 2025 | Pharmacy Education Conference Abstracts 2025 | Conference Abstract |
| 73 | Baker, J. et al. | 2025 | Clinician perspectives on parenteral nutrition (PN) in advanced cancer: Impact of experience and service design | Conference Abstract |
| 74 | Devereux, C. et al. | 2025 | "Can We Talk?" Promoting a Codeveloped Digital Resource to Support End-of-Life Conversations | Conference Abstract |
| 75 | Guccione, L. et al. | 2025 | Oral Abstracts | Conference Abstract |
| 76 | Jovic, E. et al. | 2023 | Developing a Home-based, Carer-Supported Exercise Program in Australia; Perceptions of People after Stroke and Carers on the Features of Acceptability | Conference Abstract |
| 77 | Walker, S. et al. | 2022 | Preparing for the OptiBreech Trial: a mixed methods implementation and feasibility study | Conference Abstract |
| 78 | Anonymous |  | Conference paper duplicate | Duplicate |
| 79 | Murphy, A. L. & Gardner, D. M. | 2019 | Pilot Testing the Theoretical Framework of Acceptability in a Process Evaluation of a Community Pharmacy-Based Men's Mental Health Promotion Program | Duplicate |
| 80 | McIlroy, S. et al. | 2022 | The Fibromyalgia Active Management and Exercise (FAME) programme: A service evaluation | Duplicate |
| 81 | Mendoza, G. et al. | 2024 | "They Really Need to See It." A Qualitative Study of Perspectives on Acceptability and Feasibility of Virtual Asthma Home Visiting Programs for Individuals with Poorly Controlled Asthma | Duplicate |
| 82 | Ferri, G. M. et al. | 2026 | When ICAN(S) Becomes ICAN'T: Clinician and Staff Perspectives on In-Hospital Neurotoxicity Grading | 2026 |
| 83 | Enoch, J. et al. | 2023 | Exploring patient acceptability of emerging intravitreal therapies for geographic atrophy: A mixed-methods study | Part of included study / same cohort |
| 84 | Enoch, J. et al. | 2023 | Towards a Therapy for Geographic Atrophy: A Patient's Experience | Part of included study / same cohort |
| 85 | Paynter, C. et al. | 2023 | Application of the theoretical framework of acceptability in a surgical setting: Theoretical and methodological insights | Part of included study / same cohort |
| 86 | Orkin et al. | 2025 | Perspectives of People With HIV on Implementing Long-acting Cabotegravir Plus Rilpivirine in Clinics and Community Settings in the United Kingdom: Results From the Antisexist, Antiracist, Antiageist Implementing Long-Acting Novel Antiretrovirals Study | No TFA |
