# Supplementary material for: Application of the Theoretical Framework of Acceptability in Health Research: A Scoping Review
Source: J Eval Clin Pract. 2026 Oct 27;32(7):e70698. doi: 10.1111/jep.70698 (PMC13712922; doi:10.1111/jep.70698)
Supplement: Supplementary file 3 [file JEP-32-0-s004.docx]

**Supplementary file 1 – Codebook**

| **Variable** | **Response Options** | **Definition / Decision Rule** |
| --- | --- | --- |
| **TFA application** | | |
| How TFA was Used | 1. Primary Framework 2. Post-hoc 3. Supporting Framework | 1. Primary Framework - TFA prospectively structured data collection instruments (e.g., interview guides, surveys) and was identified as the theoretical foundation in the methods. 2. Supporting Framework - TFA informed the acceptability component within a multicomponent study or shared equal status with another framework in shaping both data collection and analysis. 3. Post hoc - TFA was applied solely during analysis; data were not collected with reference to TFA constructs. |
| Framework Modified | 1. Yes 2. No | Did the authors alter the structure of TFA by adding or modifying the conceptual boundaries? This does not necessarily include redefining constructs or not using constructs for the study. |
| Acceptability Scope | 1. Acceptability only 2. Acceptability + other outcomes/frameworks (Multicomponent) | Multicomponent = a second outcome or framework was also formally assessed alongside acceptability (e.g., Feasibility, COM-B, TAM). |
| **TFA constructs** | | |
| Affective Attitude | 1. Yes 2. No 3. Unclear | How the individual feels about the intervention. |
| Burden | 1. Yes 2. No 3. Unclear | Perceived effort required to participate. |
| Ethicality | 1. Yes 2. No 3. Unclear | Extent to which the intervention fits with the individual's value system. |
| Intervention Coherence | 1. Yes 2. No 3. Unclear | Extent to which the participant understands the intervention and how it works. |
| Opportunity Costs | 1. Yes 2. No 3. Unclear | Benefits or values that must be given up engaging. |
| Perceived Effectiveness | 1. Yes 2. No 3. Unclear | Perceived likelihood the intervention will achieve its purpose. |
| Self-Efficacy | 1. Yes 2. No 3. Unclear | Confidence that participants can perform the required behaviors. |
| Coding rule (all constructs) | 1. Yes 2. No 3. Unclear | 1. Yes = any ONE of four pathways    a. Explicit reporting in Results/Discussion    b. Published instrument confirms operationalization    c. Thematic mapping onto construct definition    d. Methods confirm construct-level data collection. 2. Unclear = none of four pathways met but constructed could reasonably be inferred. 3. No = explicitly absent or authors state not assessed. |
| Emerging Constructs | Free text | Acceptability constructs proposed by authors as extending beyond the TFA seven. They are stakeholder appraisals (cognitive, or relational) or authors explicitly position the construct as extending or challenging the TFA. Constructs that are contextual, structural, or merely inductive themes are excluded |
| **Implementation context** | | |
| Implementation Stage | 1. Development/Design 2. Pre-implementation 3. During Implementation 4. Post-implementation | Operational status of the intervention at the time of the study.  Development/Design - intervention under creation or refinement.  Pre-implementation - intervention exists but has not been delivered.  During Implementation - intervention is actively being delivered, including pilots and feasibility studies.  Post implementation - intervention delivery has concluded and participants reflect retrospectively. |
| Timing of Assessment | 1. Prospective 2. Concurrent 3. Retrospective 4. Longitudinal | Timing of data collection relative to participant experience  Prospective - prior to exposure  Concurrent - during exposure  Retrospective - after exposure  Longitudinal - two or more distinct data collection points, regardless of whether the same participants are followed. |
| Implementation Setting | 1. Hospital 2. Community 3. Primary care 4. Digital/Remote 5. Home 6. School 7. Custodial facility 8. Other | Physical or organizational context of the intervention, from the participant's perspective |
| Type of Intervention | 1. Treatment - A clinical or therapeutic intervention for individuals with an existing condition, aimed at managing, alleviating, or resolving it. The object of evaluation is the treatment itself. 2. Prevention - An intervention aimed at preventing disease onset or transmission among individuals not currently affected. Includes prophylaxis, risk reduction, and health promotion activities. 3. Screening - A process for identifying unrecognized disease or risk factors among asymptomatic or at-risk individuals. The object of evaluation is the screening method or testing process. 4. Service Delivery - A model, pathway, or approach for organizing, coordinating, or providing care. The object of evaluation is how care is delivered rather than the clinical intervention itself. 5. Technology - A digital tool, platform, device, or application that is itself the intervention being evaluated, rather than merely a delivery mechanism for another intervention. 6. Training/Education - An intervention primarily intended to develop knowledge, skills, or competencies among providers, patients, caregivers, or communities. 7. Implementation strategy - A method or approach used to facilitate the adoption, integration, scale-up, or sustainability of an existing intervention in routine practice. The object of evaluation is the implementation process rather than the intervention being implemented. 8. Policy - A law, regulation, guideline, or organizational policy intended to influence service delivery, practice, or population outcomes. Distinct from implementation strategies, which focus on promoting uptake. 9. Other - Interventions that do not fit a single category above | |
| **Target Population** | | |
| Target population studied | 1. Patients 2. Service users 3. Providers 4. Healthcare workers 5. Administrators 6. 7. Community 8. Caregivers 9. Volunteers/Lay deliverers 10. Multiple | Category of target population whose acceptability was assessed. |
| Number of Target Groups | 1. One 2. Two 3. Three or more | Distinct occupational or role-defined groups. Demographic subgroups within the same role (e.g., age, sex) are not counted as separate groups. |
| **Study Methods** | | |
| Study Design | 1. Qualitative 2. Quantitative 3. Mixed methods | Overall methodological approach. |
| Data Collection | 1. Interviews 2. Surveys 3. Focus groups 4. Focus groups & interviews 5. Focus groups & surveys 6. Surveys & interviews 7. Observation & interviews 8. Focus groups, interviews & surveys 9. Other combinations | Primary method(s) used to collect acceptability data. |
| Sample Size (N) | Numeric | Total study N if acceptability is the whole study. Sub-sample N if acceptability was one component of a larger study with broader primary aims. |
